# Evaluating the Efficacy of Topical Tacrolimus Alone and in Combination with Prednisolone for Treating Subepithelial Infiltrates in Epidemic Keratoconjunctivitis

**DOI:** 10.3390/biomedicines13040895

**Published:** 2025-04-08

**Authors:** Emine Esra Karaca, Gökhan Çelik, Şule İdacı Koç, Özlem Evren Kemer

**Affiliations:** 1 Department of Ophthalmology, Ankara Bilkent City Hospital, University of Health Sciences, Ankara 06800, Turkey; gokhan.celik3@saglik.gov.tr (G.Ç.); sule.idacikoc@uhb.nhs.uk (Ş.İ.K.); ozlemevren.kemer@sbu.edu.tr (Ö.E.K.); 2 Department of Ophthalmology, Tarsus State Hospital, Mersin 33460, Turkey; 3 Department of Ophthalmology, Heartlands Hospital, University Hospitals Birmingham, Bordesley Green East, Birmingham B9 5SS, UK

**Keywords:** epidemic keratoconjunctivitis, prednisolone, subepithelial infiltrate, tacrolimus

## Abstract

**Purpose:** Epidemic keratoconjunctivitis (EKC) is a common viral ocular infection that can lead to persistent subepithelial infiltrates (SEIs), resulting in significant visual impairment and patient discomfort, necessitating effective treatment strategies beyond corticosteroid monotherapy. This study aims to evaluate the efficacy of topical tacrolimus (0.1%) ointment alone versus its combination with prednisolone (1%) drops to provide optimal therapeutic strategies for SEIs secondary to EKC. **Methods:** This retrospective observational study evaluates 102 eyes of ninety-five individuals. The patients were divided into two groups based on the treatment approach. The first group received tacrolimus ointment alone (n = 34), whereas the second group received a combination of prednisolone drops and tacrolimus ointment (n = 68). Best-corrected visual acuity (BCVA), the corneal subepithelial infiltrate score (CSIS), the subjective symptom score (SSS), and the Fantes corneal haze grading score (FCHGS) were evaluated before treatment and at 1, 3, 6, 9, 12, and 18 months post-treatment. **Results:** Both groups showed significant improvements in the BCVA, CSIS, SSS, and FCHGS values, with no significant difference between the groups at 18 months (*p* > 0.05). The combination therapy resulted in a significantly faster treatment response than tacrolimus alone (*p* < 0.05) in terms of CSIS, SSS, and FCHGS values. An increase in intraocular pressure (IOP) was observed in four patients in the combination treatment group after three months. **Conclusions:** Topical tacrolimus, both alone and in combination with topical prednisolone, was effective in treating subepithelial infiltrates secondary to EKC. Combination therapy may be applied early for faster recovery; however, close monitoring of IOP is necessary in individuals using topical prednisolone.

## 1. Introduction

Epidemic keratoconjunctivitis (EKC), which occurs approximately ten days after the onset of follicular conjunctivitis, can lead to the development of subepithelial corneal infiltrates (SEIs) [1]. SEIs are often seen in both eyes and are histologically asymmetrical. SEIs consist of aggregations of lymphocytes, histiocytes, and fibroblasts, all of which may also damage the collagen fibers of Bowman’s layer [2]. SEIs can cause significant ocular disability, resulting in decreased vision, sensitivity to light, glare, perception of halos around lights, and a foreign body sensation. These symptoms can persist for months or even years after the initial infection. Recent studies suggest that EKC remains a significant public health concern, with an estimated incidence of adenoviral conjunctivitis ranging from 20% to 40% among ocular infections globally and SEIs developing in up to 40–50% of cases, underscoring the need for effective management strategies [1,3].

Topical corticosteroid eye drops can relieve SEIs and associated symptoms. However, there is a high likelihood of recurrence when the medication is discontinued, even if it is gradually reduced. Various therapeutic modalities have been used to minimize the risks and adverse effects associated with prolonged steroid use.

Topical tacrolimus has shown promising efficacy in the treatment of SEIs following adenoviral keratoconjunctivitis (AKC), especially in cases that are resistant to corticosteroids and cyclosporine [4,5,6]. Studies have reported significant improvements in visual acuity, SEI scores, and Schirmer results with the use of topical tacrolimus, with effects lasting up to 12 months after treatment [5]. Additionally, tacrolimus has been found to be effective in reducing the number and size of SEIs, in some cases even eliminating them altogether, leading to better visual outcomes [6]. Furthermore, tacrolimus has been noted as a viable alternative to dexamethasone, with a low SEI recurrence rate and minimal side effects, making it a valuable option for managing chronic and recurrent SEIs after AKC [7]. It is known that tacrolimus demonstrates promising efficacy in modulating immune responses; however, its effects may take time to manifest. In contrast, corticosteroids provide a more rapid effect due to their potent anti-inflammatory properties. Nevertheless, the side effects associated with long-term steroid use should also be considered. Therefore, the combination of tacrolimus and steroids may enable both a rapid therapeutic response and the minimization of side effects by using lower doses of both drugs.

While topical corticosteroids offer rapid symptomatic relief for SEIs, their long-term use is limited by recurrence upon tapering and significant side effects such as IOP elevation. Tacrolimus, with its immunomodulatory properties, presents a promising alternative, yet its slower onset of action may delay recovery in severe cases. This retrospective observational study addresses the clinical gap of optimizing treatment efficacy and safety by comparing tacrolimus monotherapy with a combination of tacrolimus and prednisolone, the latter possibly providing a faster response for patients with significant visual impairment or discomfort while minimizing steroid-related risks through reduced dosing and duration.

## 2. Materials and Methods

This retrospective observational study included patients with SEI after EKC between January 2022 and December 2024. This study was conducted in compliance with the principles of the Declaration of Helsinki. In addition, ethical approval was obtained from Ankara Bilkent City Hospital first ethics committee (TABED-1/158/2024, Approval date: 24 April 2024). The informed consent was obtained from all patients.

The study population comprised 102 eyes from a total of 95 individuals who suffered from SEI due to AKC. Inclusion criteria included patients diagnosed with SEIs secondary to EKC confirmed by biomicroscopic evidence of infiltrates following a documented adenoviral infection between January 2022 and December 2024, treated with either topical tacrolimus alone or in combination with prednisolone and followed for at least 18 months. Patients with other systemic disorders or ocular pathologies, such as corneal disorders (e.g., keratoconus, existing SEI, other ocular surface diseases), ocular hypertension, glaucoma, vitreoretinal disease, ocular trauma, uveitis, amblyopia, cataract, or surgical history were excluded to ensure a homogeneous study population and minimize confounding factors that could influence the treatment outcomes of SEIs secondary to EKC. While this approach may limit the diversity of the sample, it allowed for a focused evaluation of the therapeutic effects of tacrolimus and prednisolone in an otherwise healthy ocular cohort. Furthermore, to account for prior treatments, patients with a history of topical steroid or immunosuppressive therapy within the preceding 4 weeks were excluded to minimize potential carryover effects on baseline measurements and treatment response. This ensured that the observed outcomes were attributable to the study interventions rather than residual effects of prior medications.

The patients were followed up monthly until their SEIs were resolved. Baseline and final examination measurements were obtained. Intraocular pressure (IOP) and any ocular complaints associated with the treatment were recorded at each visit to monitor adverse effects. IOP was monitored using Goldmann applanation tonometry during follow-up visits. An IOP threshold of >21 mmHg prompted intervention, with the patients prescribed brimonidine eye drops (Alphagan; Allergan Inc., Irvine, CA, USA) twice daily.

Patients’ general information (age, sex, and affected eyes) and ophthalmic examination findings, including best-corrected visual acuity (BCVA; LogMAR), IOP (mmHg), corneal subepithelial infiltrate score (CSIS), and subjective symptom score (SSS), were documented at the 1st, 3rd, 6th, 9th, 12th, and 18th months following diagnosis. CSIS values were assigned by two ophthalmologists who assessed the number of infiltrates during biomicroscopic examinations, categorizing them as follows: 0 for no infiltration, 1 for 1–5 infiltrates, 2 for six to ten infiltrates, 3 for 11–15 infiltrates, and 4 for more than 15 infiltrates. CSIS was chosen as a practical and reproducible metric based solely on the number of infiltrates observed during biomicroscopic examination, allowing for a standardized quantitative assessment across multiple time points. While this method may not capture subtle changes in infiltrate density or distribution, it was selected due to its clinical feasibility and the lack of routine availability of advanced imaging modalities, such as anterior segment optical coherence tomography (AS-OCT) or histological analysis, in our retrospective setting. The patients were asked to rate their subjective symptoms, where the SSS ranged from 0 (no symptoms) to 3 (severe symptoms). The Fantes corneal hazing grading score (FCHGS) was used to assess corneal haze caused by infiltrates, evaluated through biomicroscopic examination, with stages as follows: 0 (clear with no opacity visible by any method of microscopic slit-lamp examination), 0.5 (trace or faint haze visible only by indirect, broad tangential illumination), 1 (haze of minimal density visible with difficulty with direct or diffuse examination), 2 (mild haze easily visible with direct focal slit-lamp illumination), 3 (moderate opacity that partially obscures details of the iris), and 4 (severe opacity that completely obscures the details of intraocular structure). All tests, such as BCVA, CSIS, SSS, and FCHGS, had already been recorded in the patients’ files. Treatment success was defined as the complete resolution of SEIs (CSIS = 0) and the absence of subjective symptoms (SSS = 0), alongside the stabilization of or improvement in BCVA and FCHGS.

Statistical analyses were performed using SPSS 25.0. The mean and standard deviation values are provided for the variables. Changes in the variables before and after treatment were analyzed using a repeated measures ANOVA test with Bonferroni correction for parametric data to control for Type I error across multiple comparisons. For non-parametric data, Friedman and Wilcoxon signed-rank tests were employed, with the latter used for post-hoc pairwise comparisons to account for repeated measurements on the same subjects. These methods were selected to address potential correlations between time points and to ensure robust data interpretation, maintaining statistical power while minimizing false-positive results. Statistical significance was set at *p* < 0.05.

## 3. Results

In this study, 102 eyes from 95 individuals were included. The combined treatment group included 68 eyes from 65 individuals and the group treated with only topical tacrolimus included 34 eyes from 30 individuals. The mean age of the group treated with only topical tacrolimus was 30.88 ± 12.87 years, and that of the topical tacrolimus and prednisolone combined treatment group was 27.70 ± 10.19 years. There was no significant difference between the groups in terms of age. In the study group, 19 out of 30 patients (63.3%) were women, while in the control group, 47 out of 65 patients (72.3%) were women. A descriptive demographic analysis of the groups is presented in Table 1. The mean BCVA, IOP, CSIS, SSS, and FCHGS values of the patients in each group at baseline, first, third, sixth, ninth, twelfth, and eighteenth months and their comparative analysis are presented in Table 2.

Recurrence was observed 9 and 12 months after treatment in four patients (4.2%) who received the combined treatment; no recurrence occurred in patients who received tacrolimus alone. These recurrences were not associated with non-adherence (confirmed via patient interviews).

**Figure 1:**
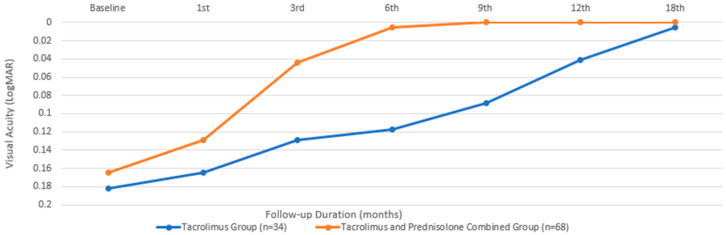
Best-corrected visual acuity changes during the treatment period.

In terms of BCVA, the baseline mean value for the group treated with only tacrolimus was 0.18 ± 0.11 LogMAR, while that for the group treated with both tacrolimus and prednisolone was 0.16 ± 0.1, with a comparable median value. There was no significant difference between the two groups in terms of baseline BCVA (*p* = 0.816). An initial significant improvement in BCVA was observed in the patients treated with tacrolimus alone at the beginning of the third month, compared to their baseline measurements (*p* = 0.024). However, in the group that received both prednisone and tacrolimus, this occurrence began during the first month (*p* = 0.003). While the combined treatment group showed a significantly faster improvement in visual performance (Figure 1), there was no significant difference between the two groups at the end of the 18th month (*p* = 0.157).

**Figure 2:**
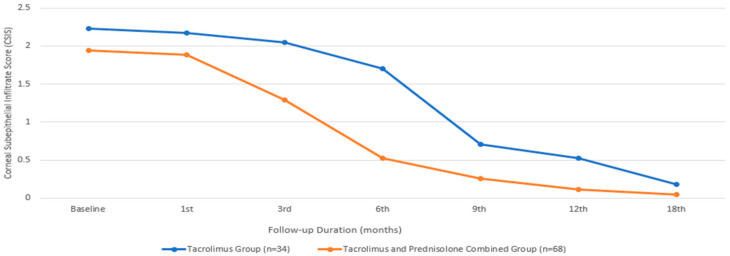
Corneal subepithelial infiltrate score changes during the treatment period.

In terms of the CSIS, the baseline mean value for the group treated with only tacrolimus was 2.23 ± 1.09, while that for the group treated with both tacrolimus and prednisolone was 1.94 ± 0.91, with a comparable median value. There was no statistically significant difference between the two groups in terms of the baseline CSIS (*p* = 0.377). An initial statistically significant improvement in the CSIS was observed in the patients treated with tacrolimus alone at the beginning of the sixth month, compared to their baseline measurements (*p* = 0.014). However, in the group receiving combined prednisone and tacrolimus, this occurrence began during the third month (*p* ≤0.001). While the combined treatment group showed a significantly faster improvement in visual CSIS (Figure 2), there was no statistically significant difference between the two groups at the end of the 18th month (*p* = 0.187).

**Figure 3:**
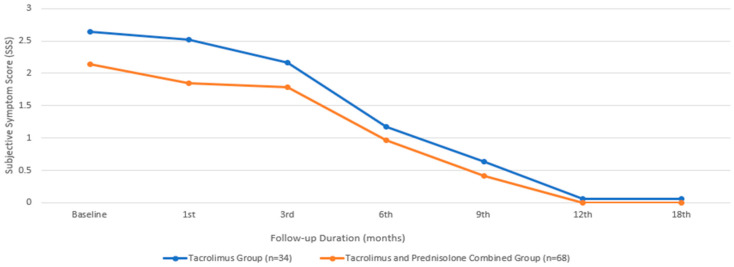
Subjective symptom score changes during the treatment period.

In terms of SSS, the baseline mean value for the group treated with only tacrolimus was 2.64 ± 0.49, while that for the group treated with both tacrolimus and prednisolone was 2.14 ± 0.89, with a comparable median value. There was no statistically significant difference between the two groups in terms of the baseline SSS (*p* = 0.065). An initial statistically significant improvement in the SSS was observed in the patients treated with tacrolimus alone at the beginning of the third month, compared to their baseline measurements (*p* = 0.011). However, in the group that received combined prednisolone and tacrolimus, this occurrence began during the first month (*p* = 0.002). While the combined treatment group showed a significantly faster improvement in SSS (Figure 3), there was no statistically significant difference between the two groups at the end of the 18th month (*p* = 1.00).

**Figure 4:**
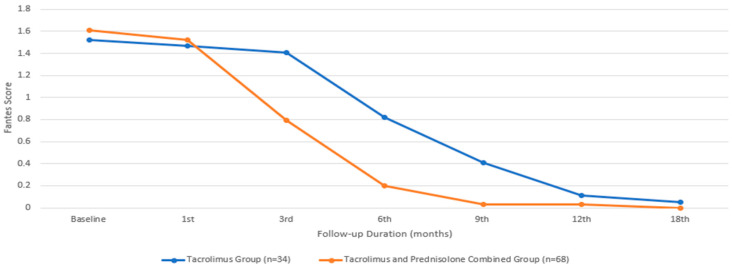
Fantes corneal haze grading score changes during the treatment period.

**Figure 5:**
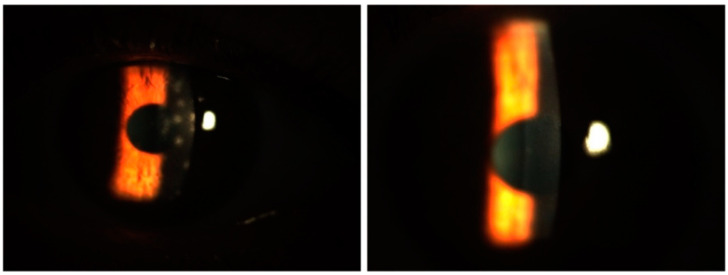
Anterior segment photos from baseline (**left**) and three months after tacrolimus-only ointment treatment (**right**) in a patient.

In terms of the Fantes score, the baseline mean value for the group treated with only tacrolimus was 1.52 ± 0.51, while that for the group treated with both tacrolimus and prednisolone was 1.61 ± 0.69, with a comparable median value. There was no statistically significant difference between the two groups in terms of the baseline Fantes score (*p* = 0.065). An initial statistically significant improvement in the Fantes score was observed in the patients treated with tacrolimus alone at the beginning of the third month, compared to their baseline measurements (*p* = 0.011). However, in the group that received combined prednisone and tacrolimus, this improvement began during the first month (*p* = 0.002). While the combined treatment group showed a significantly faster improvement in the Fantes score (Figure 4), there was no statistically significant difference between the two groups at the end of the 18th month (*p* = 1.00). Figure 5 depicts an anterior segment photo from baseline and one from three months after tacrolimus ointment treatment, demonstrating a reduction in corneal haze and infiltrate density. Due to the retrospective nature of this study, additional comparative images between the tacrolimus-only and combination therapy groups were not available. However, the statistical improvements in CSIS and FCHGS (Figure 2 and Figure 4) corroborate the clinical resolution observed here.

**Figure 6:**
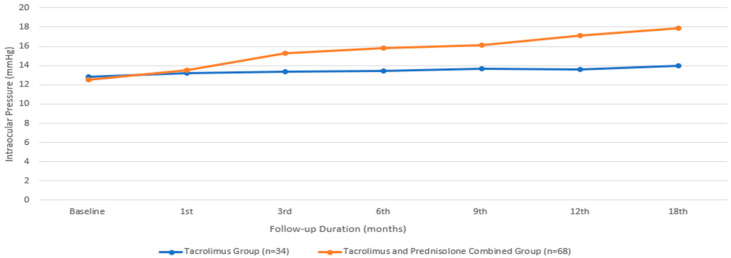
Intraocular pressure changes during the treatment period.

Regarding IOP, the median value for the group treated with only tacrolimus was 12.82 ± 1.77 mmHg, while that for the group treated with both tacrolimus and prednisolone had a comparable mean value of 12.5 ± 2.63 mmHg. There was no statistically significant difference between the two groups in terms of the baseline IOP (*p* = 0.371). There was no statistically significant difference in IOP among the individuals who received only tacrolimus during the therapy period (*p* = 0.609, according to the Friedman test). However, in the group receiving the combined treatment, IOP showed a statistically significant increase from the baseline in the third month (*p* = 0.001). The IOP of the group receiving combined treatment was still higher at the end of the 18th month compared to the group receiving only the tacrolimus treatment (*p* ≤ 0.001) (Figure 6). Four patients who underwent the combined treatment experienced an elevation in IOP that required treatment. Brimonidine eye drops (Alphagan; Allergan Inc., Irvine, CA, USA) were prescribed for twice-daily use. After brimonidine administration, the IOP values remained within normal limits.

Adverse events related to tacrolimus included a brief burning sensation and eye watering in two of the 34 patients (5.9%) in the tacrolimus-only group and in six of the 68 patients (8.8%) in the combination group, occurring within minutes of application, with all patients tolerating continued use. Six additional patients in the combination group (8.8%) discontinued tacrolimus due to persistent discomfort, burning, and redness. To mitigate these effects, patients were advised to apply the ointment at bedtime and use artificial tears prior to application, an approach that improved tolerance in most cases.

During the therapy, the parameters within the groups and their fluctuations were assessed using Friedman analysis, followed by the Wilcoxon ranked test. The z- and *p*-values for this study are listed in Table 3.

## 4. Discussion

SEIs that occur after AKC can be challenging for patients, as they can persist for a long time, even months or years after the initial infection. This can result in considerable corneal damage. The chronic nature of the disease is frequently exacerbated by prolonged treatments, as there is currently no effective antiviral medication available for treating adenovirus and its complications. This limitation has prompted investigations into other methods of treatment. For instance, a study revealed that the use of PVP-iodine or PVA-iodine, either alone or in combination with a steroid, is linked to a reduced likelihood of developing SEIs compared with the use of artificial tears or steroids (based on data of very low confidence) [3].

The use of topical steroids for the treatment of SEIs remains controversial. Ophthalmologists often prescribe them during the acute period; however, their relieving impact may only be temporary. In addition, the duration of the disease and infection may be extended by the application of steroids owing to an elevated rate of adenovirus replication and a prolonged period of viral shedding, as evidenced in studies conducted on animal models [8]. Corticosteroids are believed to hinder the ability of the immune system due to their anti-inflammatory and immunosuppressive properties. Furthermore, steroids have a tendency to induce significant adverse effects, such as an increase in IOP and the development of cataracts [9,10]. As a result, other medications in addition to steroids may be required for therapy. The observed elevation in IOP in the combined treatment group (T+P) from the third month onward (*p* = 0.001) highlights a known side effect of prolonged corticosteroid use. This finding aligns with the exclusion of patients with pre-existing glaucoma or other conditions predisposing to IOP elevation, as outlined in our criteria. The absence of significant IOP changes in the tacrolimus-only group (*p* = 0.609) suggests that tacrolimus may offer a safer profile for long-term management of SEIs, particularly in patients at risk of developing steroid-induced ocular hypertension. The regular monitoring of IOP ensured the timely detection of elevations, with a predefined intervention threshold of >21 mmHg triggering the use of IOP-lowering agents. This protocol effectively managed steroid-induced IOP increases, suggesting that combination therapy can be safely administered with rigorous follow-up. Regarding cataract development, unlike studies reporting cataract formation with prolonged steroid use [8], our cohort showed no such complications, possibly due to the limited steroid duration and close IOP monitoring. Regarding disease recurrence, the recurrence observed in the combination group warrants further consideration. As these cases were unrelated to IOP elevation or non-adherence, they may reflect a rebound inflammatory response following steroid withdrawal, a phenomenon noted in prior studies [8]. This suggests that gradual tapering and prolonged tacrolimus use post-steroid cessation could mitigate recurrence risk.

Topical cyclosporin (CsA) is the first substance considered to provide anti-inflammatory properties similar to those of topical steroids while also diminishing the adverse effects of steroids [11]. Owing to its ability to decrease inflammation on the anterior ocular surface, it is considered an effective treatment choice for both adenoviral conjunctivitis and vernal conjunctivitis [12]. Another study found that tacrolimus 0.1% ointment demonstrated significant efficacy in reducing eye discomfort and reliance on local corticosteroids in patients with atopic keratoconjunctivitis, without affecting visual acuity. This retrospective study highlighted its potential as an effective and well-tolerated treatment option for cases unresponsive to first-line therapies [13]. Levinger et al. [14] administered 1% CsA eye drops twice a day to nine individuals who were resistant to steroids or responded to steroids. A total of 66% of the patients showed clinical improvement, while 34% maintained stability. Okumus et al. [15] showed that topical CsA treatment provided clinical improvement, as observed in 18 eyes (81.9%), while four eyes (18.1%) exhibited a decrease in SEIs that did not completely resolve during the treatment period. In another study, symptomatic SEIs occurred following AKC in 12 eyes of seven patients who responded to corticosteroid eye drops but were resistant to dosage reduction. The authors proposed that corticosteroid eye drops may be reduced after commencing CsA eye drops [16].

Topical tacrolimus, like CsA, is regarded as an efficacious therapeutic choice for both adenoviral conjunctivitis and vernal conjunctivitis because of its capacity to diminish inflammation on the anterior surface of the eye [17]. Levinger et al. [14] demonstrated that the use of topical tacrolimus 0.03% was both safe and effective in treating a limited number of individuals with SEIs. Their study showed a notable enhancement in the average logMAR BCVA of the group, equivalent to approximately two Snellen lines, with a *p*-value of 0.042, after the 22-week treatment period. None of these patients had photopsia, foreign body feeling, or any other ocular adverse effects commonly associated with the use of topical tacrolimus.

In another investigation that demonstrated the efficacy of tacrolimus in the treatment of steroid-resistant SEIs, all patients were started on 0.03% tacrolimus, along with 0.5% loteprednol etabonate or 1% prednisolone. The majority of patients (85.7%) were able to tolerate tacrolimus, and all of them experienced a reduction in infiltrates and an improvement in CDVA and other ocular symptoms [18]. A recent study compared the safety and effectiveness of tacrolimus 0.03% ointment and 0.05% dexamethasone ointment for the treatment of SEIs. The study found that tacrolimus 0.03% was a viable alternative to dexamethasone 0.05%, with a low relapse rate. However, it may cause a burning sensation and foreign body sensation in certain patients [7]. In another investigation of SEI cases that were resistant to topical steroid drops, the number and extent of SEIs were reduced in 62.35% of the eyes, while they disappeared in 31.76%. The patients’ visual acuity improved significantly following treatment, with highly statistically significant differences. Overall, the tolerance was satisfactory, with the eye drop group demonstrating superior tolerance [6].

Arici et al. [5] implemented topical tacrolimus treatment for cases that were resistant to topical steroid and CsA treatment. The study included 15 eyes from 11 patients with SEIs and 16 eyes from 16 healthy controls. The study group exhibited substantial enhancements in the BCVA, CSIS, FCHGS, and Schirmer results starting from the 3-month visit, and these enhancements persisted for the duration of the 12-month treatment period. Despite corticosteroid and/or CsA treatment, topical 0.03% tacrolimus may demonstrate efficacy against SEIs that persist for a minimum of two years, without any significant adverse effects. Although considerable improvements in the BCVA, SEIs, and Schirmer results required a minimum of three months of treatment, a decrease in the Oxford score took a minimum of six months. Compared to our findings, this suggests that both tacrolimus alone and in combination with prednisolone offer comparable long-term visual outcomes, with BCVA stabilizing at 0.05 ± 0.02 LogMAR and 0 LogMAR, respectively, by 18 months. For instance, Arici et al. reported sustained BCVA improvements over 12 months with tacrolimus in steroid-resistant cases, though with slower initial responses. However, our combination therapy group exhibited faster symptom resolution, potentially improving quality of life earlier in the treatment course [5].

The faster improvement observed with the combination therapy suggests its potential as a first-line option for severe SEIs, where rapid symptom relief and visual recovery are critical. However, given the risk of IOP elevation, it may be best reserved for cases with significant visual impairment or debilitating symptoms, while tacrolimus monotherapy could suffice for milder presentations. Clinicians should weigh these benefits against the need for close IOP monitoring or disease severity when selecting this approach.

This study has some limitations. First, the sample size was relatively small, a factor that may affect the applicability of the findings. Another limitation of this study is the reliance on CSIS, which quantifies infiltrates based solely on their number rather than their density, size, or distribution. Incorporating advanced imaging techniques, such as AS-OCT, or histological analysis could provide a more comprehensive understanding of infiltrate dynamics and their response to treatment. However, these methods were not feasible in this retrospective study due to resource constraints and the lack of standardized protocols for such assessments at the time of data collection. On the other hands, the extensive exclusion criteria employed in this study, while ensuring a controlled evaluation of treatment efficacy, may limit the generalizability of our findings to a broader clinical population. Patients with EKC often present with comorbidities such as diabetes, autoimmune disorders, or concurrent ocular conditions, which could alter the response to topical tacrolimus or prednisolone. Future studies including a more diverse patient population with comorbidities are warranted to validate the applicability of these findings in real-world clinical settings.

## 5. Conclusions

This study demonstrates that topical tacrolimus, either alone or in combination with prednisolone, effectively reduces SEIs secondary to EKC, improving BCVA, CSIS, SSS, and FCHGS over 18 months. The combination therapy offers a significantly faster therapeutic response (*p* < 0.05), which may be advantageous for patients requiring rapid symptom relief. However, the increased IOP observed in the combination group (*p* < 0.001) underscores the need for close monitoring, particularly in patients susceptible to steroid-induced ocular hypertension. Tacrolimus alone presents a viable alternative with a lower risk of IOP elevation and no recurrence, though its slower onset may limit its use in acute settings. Future prospective studies with larger sample sizes are recommended to validate these findings and explore optimal dosing regimens to balance efficacy and safety.

## Figures and Tables

**Table 1:** Demographic and descriptive characteristics of the study population.

	Only T Group	T+P Group	*p*-Value
Age (years, mean ± SD)	30.88 ± 12.87	27.70 ± 10.19	0.136
Sex (Female, n, %)	19 (63.3%)	47 (72.3%)	0.374
BCVA (logMAR)	0.18 ± 0.11	0.16 ± 0.11	0.816
IOP (mmHg)	12.82 ± 1.77	12.5 ± 2.63	0.371
CSIS	2.23 ± 1.09	1.94 ± 0.91	0.377
SSS	2.64 ± 0.49	2.14 ± 0.89	0.065
FCHGS	1.52 ± 0.51	1.61 ± 0.69	0.832

**Table 2:** Descriptive analysis and comparison of variables between groups (only T group: tacrolimus alone; T+P group: tacrolimus + prednisolone; mean ± SD; bold *p*-values indicate statistical significance, *p* < 0.05).

Variable	Time Point	Only T Group (n = 34)	T+P Group (n = 68)	*p*-Value
BCVA (logMAR)	Baseline	0.18 ± 0.11	0.16 ± 0.11	0.816
	First Month	0.16 ± 0.09	0.12 ± 0.07	0.107
	Third Month	0.12 ± 0.09	0.04 ± 0.06	**<0.001**
	Sixth Month	0.11 ± 0.06	0.005 ± 0.023	**<0.001**
	Ninth Month	0.08 ± 0.06	0	**<0.001**
	Twelfth Month	0.04 ± 0.05	0	**<0.001**
	Eighteenth Month	0.05 ± 0.02	0	0.157
IOP (mmHg)	Baseline	12.82 ± 1.77	12.5 ± 2.63	0.371
	First Month	13.23 ± 2.33	13.5 ± 2.98	0.976
	Third Month	13.35 ± 1.76	15.26 ± 3.33	0.054
	Sixth Month	13.47 ± 2.37	15.79 ± 3.40	**0.016**
	Ninth Month	13.64 ± 2.20	16.11 ± 3.17	**0.005**
	Twelfth Month	13.58 ± 2.09	17.11 ± 3.03	**<0.001**
	Eighteenth Month	13.94 ± 2.30	17.88 ± 3.57	**<0.001**
CSIS	Baseline	2.23 ± 1.09	1.94 ± 0.91	0.377
	First Month	2.17 ± 1.07	1.88 ± 0.91	0.363
	Third Month	2.05 ± 0.96	1.29 ± 1.11	**0.010**
	Sixth Month	1.70 ± 0.68	0.52 ± 0.86	**<0.001**
	Ninth Month	0.70 ± 0.77	0.26 ± 0.66	**0.011**
	Twelfth Month	0.52 ± 0.71	0.11 ± 0.32	**0.013**
	Eighteenth Month	0.17 ± 0.39	0.05 ± 0.23	0.187
SSS	Baseline	2.64 ± 0.49	2.14 ± 0.89	0.065
	First Month	2.52 ± 0.62	1.85 ± 0.82	**0.005**
	Third Month	2.17 ± 0.72	1.79 ± 0.76	0.100
	Sixth Month	1.17 ± 0.63	0.97 ± 0.62	0.271
	Ninth Month	0.64 ± 0.70	0.41 ± 0.49	0.273
	Twelfth Month	0.05 ± 0.24	0	0.157
	Eighteenth Month	0.05 ± 0.24	0	0.157
FCHGS	Baseline	1.52 ± 0.51	1.61 ± 0.69	0.832
	First Month	1.47 ± 0.51	1.52 ± 0.70	0.982
	Third Month	1.41 ± 0.50	0.79 ± 0.68	**0.003**
	Sixth Month	0.82 ± 0.52	0.20 ± 0.41	**<0.001**
	Ninth Month	0.41 ± 0.50	0.02 ± 0.17	**<0.001**
	Twelfth Month	0.11 ± 0.33	0.02 ± 0.17	0.211
	Eighteenth Month	0.05 ± 0.24	0	0.157

**Table 3:** Wilcoxon test for the comparison of the mean LogMAR BCVA, CSIS, SSS, and FCHGS at the baseline visit and at the 1-month, 3-month, 6-month, 9-month, 12-month, and 18-month visits for each group. ‘Only T group’ refers to the group treated only with tacrolimus; ‘T+P group’ refers to the combined treatment group. Bold *p*-values indicate statistical significance, *p* < 0.05.

	Only T Group (n = 34)		T+P Combined Group (n = 68)	
	z value	*p* value	z value	*p* value
BCVA Baseline—First Month	−1.342	0.180	−2.972	**0.003**
BCVA Baseline—Third Month	−2.264	**0.024**	−4.770	**<0.001**
BCVA Baseline—Sixth Month	−2.414	**0.016**	−5.135	**<0.001**
BCVA Baseline—Ninth Month	−3.213	**0.001**	−5.136	**<0.001**
BCVA Baseline—Twelve Month	−3.824	**<0.001**	−5.136	**<0.001**
BCVA Baseline—Eighteenth Month	−3.711	**<0.001**	−5.136	**<0.001**
CSIS Baseline—First Month	−1.000	0.317	−1.414	0.157
CSIS Baseline—Third Month	−1.732	0.083	−4.119	**<0.001**
CSIS Baseline—Sixth Month	−2.460	**0.014**	−5.295	**<0.001**
CSIS Baseline—Ninth Month	−3.720	**<0.001**	−5.188	**<0.001**
CSIS Baseline—Twelve Month	−3.685	**<0.001**	−5.170	**<0.001**
CSIS Baseline—Eighteenth Month	−3.668	**<0.001**	−5.159	**<0.001**
SSS Baseline—First Month	−1.414	0.157	−3.162	**0.002**
SSS Baseline—Third Month	−2.530	**0.011**	−3.464	**0.001**
SSS Baseline—Sixth Month	−3.601	**<0.001**	−4.875	**<0.001**
SSS Baseline—Ninth Month	−3.695	**<0.001**	−4.856	**<0.001**
SSS Baseline—Twelve Month	−3.739	**<0.001**	−5.177	**<0.001**
SSS Baseline—Eighteenth Month	−3.739	**<0.001**	−5.177	**<0.001**
FCHGS Baseline—First Month	−1.000	0.317	−1.732	0.083
FCHGS Baseline—Third Month	−1.414	0.157	−5.112	**<0.001**
FCHGS Baseline—Sixth Month	−3.207	**0.001**	−5.169	**<0.001**
FCHGS Baseline—Ninth Month	−3.578	**<0.001**	−5.209	**<0.001**
FCHGS Baseline—Twelve Month	−3.619	**<0.001**	−5.209	**<0.001**
FCHGS Baseline—Eighteenth Month	**−3.729**	**<0.001**	**−5.201**	**<0.001**

## Data Availability

Dataset can be shared uponn editoral board request.
